# A Missed Celiac Artery Aneurysm Leading to Rupture: A Case Report

**DOI:** 10.5811/cpcem.2020.6.46513

**Published:** 2020-07-14

**Authors:** Jason Della Vecchia, Eric Blazar

**Affiliations:** * Clovis Community Medical Center, Department of Emergency Medicine, Clovis, California; † Inspira Medical Center, Department of Emergency Medicine, Vineland, New Jersey

**Keywords:** celiac artery aneurysm, abdominal pain, neurofibromatosis

## Abstract

**Introduction:**

Abdominal pain is a common complaint seen in the emergency department (ED). We report a case of celiac artery aneurysm (CAA) in a male patient presenting with abdominal pain to the ED on two separate occasions, approximately 24 hours apart.

**Case Report:**

On the initial visit the patient was discharged with undifferentiated abdominal pain after computed tomography imaging and laboratory investigations. On the repeat visit he was found to have a rapidly expanding CAA with rupture. He became unstable requiring intubation, blood transfusions, and emergent transfer to a tertiary care center for surgical management where, unfortunately, he died hours after failed operative management.

**Conclusion:**

Although rare, abdominal pain caused by CAAs can rapidly progress to rupture and have a high mortality.

## INTRODUCTION

Abdominal pain is a common presenting complaint in the emergency department (ED) with a large differential diagnosis list ranging from non-emergent to emergent life-threatening diagnoses. We present a case of a 41-year-old male who initially presented to the ED with abdominal pain and a stable-appearing neurofibroma adjacent to the celiac artery on work-up. On a repeat visit the next day, the patient was found to have a rapidly expanding celiac artery aneurysm (CAA) with rupture.

## CASE REPORT

### Initial Visit

A 41-year-old male presented to the ED for worsening abdominal pain over the prior three days. The patient described the pain as intermittent, sharp, and crampy in the upper quadrants. He reported constipation but denied fevers, nausea, vomiting, or diarrhea. He reported a past medical history of hypertension and neurofibromatosis. He denied prior surgeries, smoking, alcohol abuse, or illicit drug use.

The vital signs were heart rate 94 beats per minute (bpm); respiratory rate 18 breaths per minute; blood pressure 159/90 millimeters of mercury (mmHg); temperature 36.5 degrees Celsius; and oxygen saturation 98% on room air. The patient appeared comfortable, but his physical exam was remarkable for tenderness in the upper abdomen without rebound or guarding. Murphy’s sign was negative, and no masses were palpated The remainder of his physical exam was unremarkable. The emergency physician ordered a complete blood count (CBC), comprehensive metabolic panel (CMP), lipase, urinalysis (UA), and computed tomography (CT) of the abdomen and pelvis with intravenous (IV) contrast. CBC, CMP, lipase and UA were within normal limits.

The CT revealed no acute intra-abdominal process but revealed a stable, round focus of low attenuation adjacent to the celiac artery. This was thought to be a neurofibroma given the patient’s history and stable appearance from a CT performed nine years prior. The diameter of the opacified celiac artery was also similar to the prior study at 1.2 centimeters (cm). The patient was treated with one liter normal saline bolus and famotidine. On re-evaluation he was symptom free and informed of the results of the work-up that was performed. He was diagnosed with undifferentiated abdominal pain and counseled to return to the ED for worsening pain, the development of fever, uncontrollable vomiting, or any new concerns.

### Repeat Visit

The patient returned to the ED the next day for acute worsening of his pain that became diffuse and constant. He reported several episodes of non-bloody vomiting and several episodes of non-bloody diarrhea after taking milk of magnesia and a Fleet enema for his constipation. The vital signs were heart rate 75 bpm; respiratory rate 18 breaths per minute; blood pressure 170/95 mmHg; temperature 36.2 degrees Celsius; and oxygen saturation 100% on room air. The patient appeared very uncomfortable and was writhing in pain. He had diffuse tenderness on abdominal exam, but no palpable pulsatile masses or auscultated abdominal bruits. He had good distal perfusion to his extremities with distal pulses equal. Otherwise, his physical exam was unremarkable.

**Image 1:**
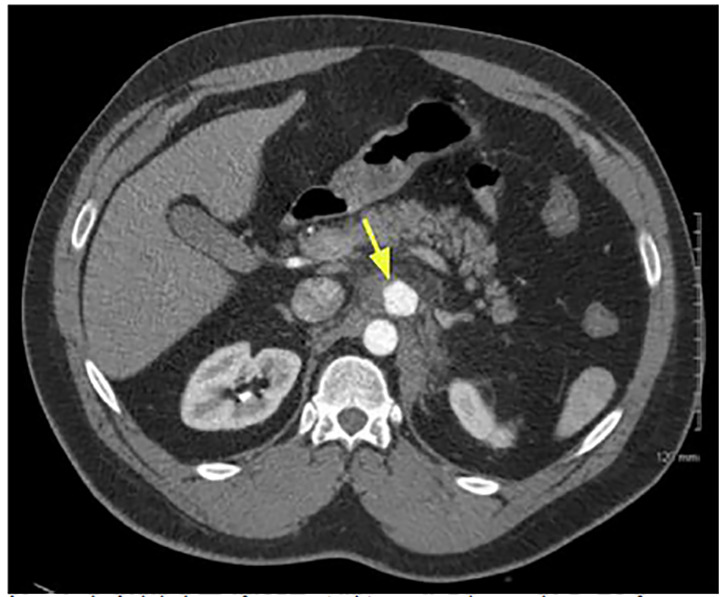
Axial view of computed tomography angiogram of abdomen and pelvis showing a proximal celiac artery aneurysm (arrow) with surrounding inflammation and trace hemorrhage.

**Image 2:**
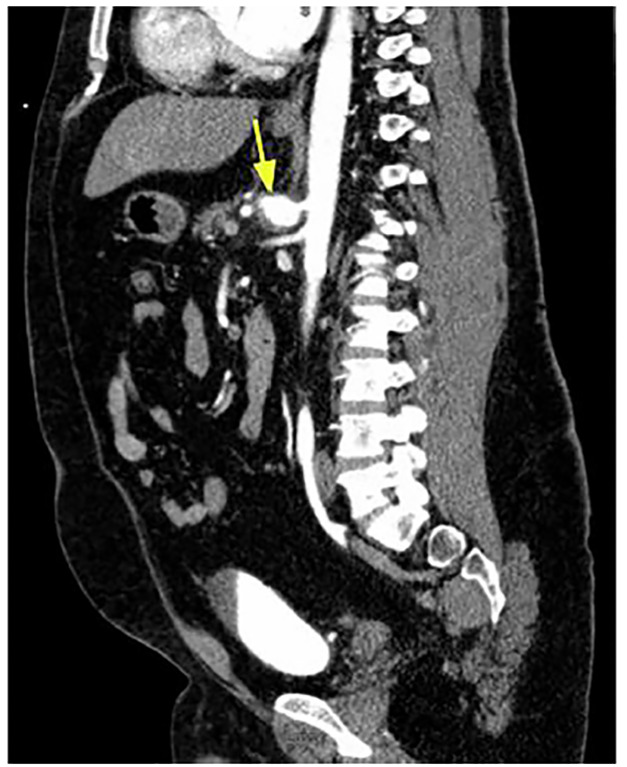
Sagittal view of computed tomography angiogram of abdomen and pelvis showing a proximal celiac artery aneurysm (arrow) with surrounding inflammation and trace hemorrhage.

CBC, CMP, lipase, lactic acid, and a CT angiogram of the abdomen and pelvis were ordered, along with IV opioid and fluids. The patient received multiple doses of opiate analgesia, including hydromorphone, without relief. CBC was remarkable for leukocytosis of 17.3 x 10^9^ per liter (L) (normal range: 4.5 to 11.0 x 10^9^/L), and lactic acid was 2.0 millimoles per liter (mmol/L) (normal range: 0.5 to 2.0 mmol/L). CMP and lipase were unremarkable. The CT angiogram of the abdomen and pelvis (Images 1 and 2) revealed a 3.2 x 2.4 cm proximal CAA with surrounding inflammatory change and trace hemorrhage. The remaining vasculature was unremarkable.

**CPC-EM Capsule**

What do we already know about this clinical entity?

Celiac artery aneurysm (CAA) is an uncommon vascular lesion that can rapidly expand and rupture.

What makes this presentation of disease reportable?

Symptomatic CAA can present with non-specific abdominal pain and be missed on initial imaging.

What is the major learning point?

Failure to consider a diagnosis of CAA in a differential can lead to a failure to diagnose this potentially life-threatening condition.

How might this improve emergency medicine practice?

Emergency physicians should consider this rare life-threatening diagnosis and be skeptical of all diagnostic tests.

Upon return from CT, the patient’s status deteriorated. He became diaphoretic, tachycardic, and hypotensive. Point-of-care ultrasound revealed fluid in Morrison’s pouch. Central venous access was obtained and the patient was stabilized with four units of blood. He was transferred to a tertiary care facility for emergent surgical repair. The patient was brought to the operating room emergently at the tertiary care facility and underwent exploratory laparotomy. He was found to have a large retroperitoneal hematoma from a bleeding CAA. Both proximal and distal control was obtained, and the CAA was ligated.

Intraoperatively he had an estimated blood loss of eight liters and received 20 units of blood by massive transfusion protocol. He developed a coagulopathy, and the bleeding could not be controlled. The area of bleeding was packed and the patient transferred to the post-anesthesia care unit with an open abdomen, vacuum-assisted wound closure for continued resuscitation. The patient had a do-not-resuscitate order placed by family and expired a few hours after surgery.

## DISCUSSION

CAAs are uncommon vascular lesions, accounting for 5.1% of all splanchnic artery aneurysms, which have an incidence ranging from 0.1–2% in the adult population.^1^ The etiology of CAA includes infectious diseases, atherosclerosis, trauma, or congenital diseases. While infectious diseases such as syphilis and tuberculosis were once the most common causes, today atherosclerosis is more common.^2^ Patients can present with vague abdominal or back pain representing an expanding hematoma that may eventually rupture into the peritoneum, retroperitoneum, or the thorax, leading to an unstable patient. However, the majority of patients will be asymptomatic and the aneurysm is found incidentally on imaging or angiography in search or treatment of other diagnoses. Unlike abdominal aortic aneurysms (AAA), the natural history and management of CAAs is fairly unknown given their low prevalence compared to AAAs; however, like AAAs the rupture carries a high rate of mortality.^1^ In the largest known case series with 18 patients, the two patients with rupture died.^3^

With the increase in CT imaging in the ED, it can be expected that incidental CAAs will be found. Although there are no clear consensus guidelines, it is recommended that surgical repair be done on aneurysms that are symptomatic, greater in size than two cm, those that expand greater than 0.5 cm per year, or those found in asymptomatic women who are either pregnant or of chilbearing age.^4^ The treatment approach varies depending on the preference of the vascular surgeon, but it can involve an open procedure with celiac artery ligation with or without revascularization, or endovascular approaches with stenting and embolization. On the initial visit for our patient a vascular origin of his pain was not considered given the stable CT findings and no report to suggest an aneurysm in the radiologist’s differential for the abnormality seen adjacent to the celiac artery. Unfortunately, this misdiagnosis led to a delay in diagnosis and likely contributed to the patient’s mortality on his repeat visit.

## CONCLUSION

Celiac artery aneurysms can be asymptomatic but have the potential to be life-threatening if presenting with rupture. Although rare, it is important for emergency physicians to be aware of this diagnosis and refer patients for early treatment if found on imaging.

## References

[1] Pulli R, Dorigo W, Troisi N (2008). Surgical treatment of visceral artery aneurysms: a 25-year experience. J Vasc Surg.

[2] Takeuchi N, Soneda J, Naito H (2017). Successfully-treated asymptomatic celiac artery aneurysm: a case report. Int J Surg Case Rep.

[3] Stone W, Abbas M, Gloviczki P (2002). Celiac arterial aneurysms. Archives of Surg.

[4] Azimi-Ghomi O, Khan K, Ulloa K (2017). Celiac artery aneurysm diagnosis and repair in the postpartum female. J Surg Case Rep.

